# The centre of rotation of the shoulder complex and the effect of normalisation

**DOI:** 10.1016/j.jbiomech.2016.03.035

**Published:** 2016-06-14

**Authors:** Celia Amabile, Anthony M.J. Bull, Angela E. Kedgley

**Affiliations:** Department of Bioengineering, Imperial College London, London, United Kingdom

**Keywords:** Centre of rotation, Shoulder, Normalisation

## Abstract

Shoulder motions consist of a composite movement of three joints and one pseudo-joint, which together dictate the humerothoracic motion. The purpose of this work was to quantify the location of the centre of rotation (CoR) of the shoulder complex as a whole. Dynamic motion of 12 participants was recorded using optical motion tracking during coronal, scapular and sagittal plane elevation. The instantaneous CoR was found for each angle of elevation using helical axes projected onto the three planes of motion. The location of an average CoR for each plane was evaluated using digitised and anthropometric measures for normalisation. When conducting motion in the coronal, scapular, and sagittal planes, respectively, the coefficients for locating the CoRs of the shoulder complex are −61%, −61%, and −65% of the anterior–posterior dimension – the vector between the midpoint of the incisura jugularis and the xiphoid process and the midpoint of the seventh cervical vertebra and the eighth thoracic vertebra; 0%, −1%, and −2% of the superior–inferior dimension – the vector between the midpoint of the acromioclavicular joints and the midpoint of the anterior superior iliac spines; and 57%, 57%, and 78% of the medial–lateral dimension −0.129 times the height of the participant. Knowing the location of the CoR of the shoulder complex as a whole enables improved participant positioning for evaluation and rehabilitation activities that involve movement of the hand with a fixed radius, such as those that employ isokinetic dynamometers.

## 1 Introduction

The shoulder complex consists of four joints (glenohumeral, acromioclavicular, sternoclavicular, and scapulothoracic) that act together to enable its full range of motion (RoM). Previous research has focused primarily on the rotation of the glenohumeral joint alone (Campbell et al., 2009, Hill et al., 2007, Lempereur et al., 2010, Lempereur et al., 2011, Monnet et al., 2007, Stokdijk et al., 2000, Veeger, 2000). However, rotation of the humerus at the glenohumeral joint does not occur in isolation; in a pair of studies, Walmsley examined the movement of the position of the glenohumeral joint while using a dynamometer, relative to a laboratory reference frame, finding it to be of the order of several centimeters (Walmsley, 1993a, Walmsley, 1993b). Other groups have examined scapular kinematics in isolation (Matsuki et al., 2011) and the scapulohumeral rhythm (Yoshizaki et al., 2009), but none have quantified the location of the centre of rotation (CoR) of the entire shoulder complex.

The position of the joint CoR is important when considering subject positioning for evaluation and rehabilitation activities. For example, use of isokinetic dynamometers to assess strength at, and perform rehabilitation of a given joint, partly depends on the ability to align the dynamometer with the joint CoR, which may not be the same as the geometric centre of the joint. Incorrect alignment will result in pain and potential injury to the subject (Codine et al., 2005), as well as inaccurate outputs. Prior studies reported on the difficulty of aligning subjects due to the unknown location of the shoulder complex CoR relative to the thorax (Shklar and Dvir, 1995). Determining its location would facilitate more effective evaluation of the strength of the shoulder complex and improved positioning for rehabilitation. However, as the shoulder complex is not a single joint, the CoR cannot simply be estimated visually. Therefore, the aim of this work was to quantify the location of the CoR of the complete shoulder complex relative to the thorax.

Given the location of such a point, the objectives were to assess the inter- and intra-subject repeatability (*S*_inter_ and *S*_intra_, respectively) of this point׳s position, determine the method of normalisation that best estimated the CoR of the shoulder complex for each plane of motion studied, and quantify how the error in locating this point varied during arm elevation.

## 2 Materials and methods

### 2.1 Participants

Twelve volunteers (four women, eight men; age 26.4±5.6 years old; height 1.76±0.11 m; weight 71.4±10.7 kg; BMI 22.8±2.1 kg/m^2^) participated in the study that was approved by the institutional ethics committee. All participants gave informed written consent prior to testing and were screened to ensure they had no previous surgery, injury or chronic pain in either shoulder. Laterality was assessed with a modified Edinburgh Inventory Handedness Score (Milenkovic and Dragovic, 2013).

### 2.2 Experimental protocol

**Fig. 1:**
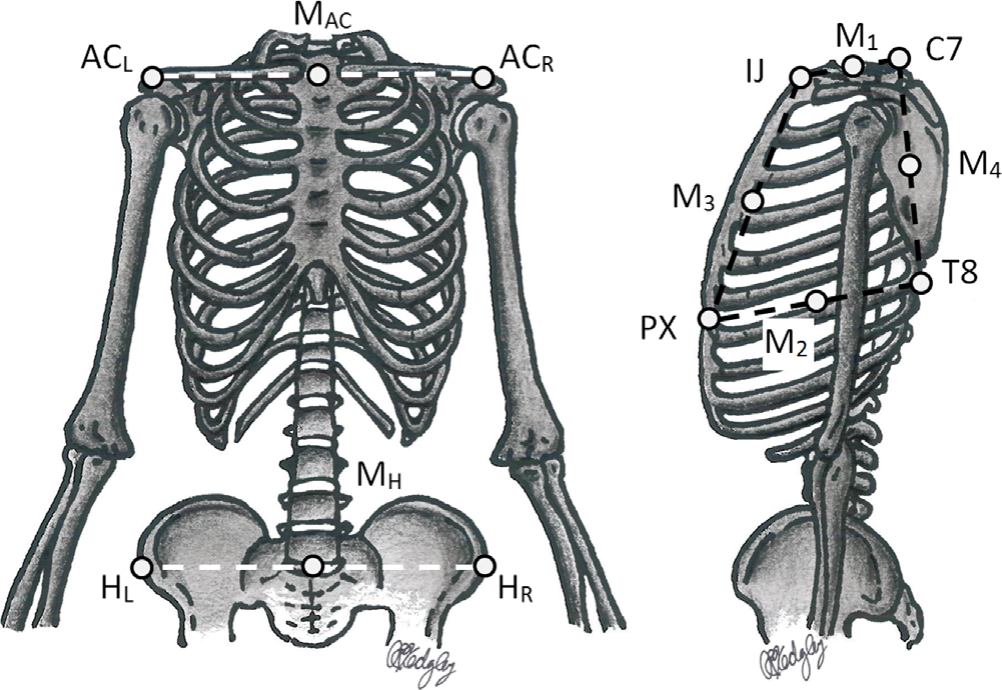
Anatomic and derived landmarks used for the four methods of normalisation. These are the left acromioclavicular joint (AC_L_), right acromioclavicular joint (AC_R_), midpoint between AC_L_ and AC_R_ (M_AC_), midpoint between the two anterior superior iliac spines (H_L_ and H_R_) (M_H_), midpoint between the incisura jugularis (IJ) and seventh cervical vertebra (C7) (M_1_), midpoint between the xiphoid process (PX) and eighth thoracic vertebra (T8) (M_2_), midpoint between IJ and PX (M_3_), and midpoint between C7 and T8 (M_4_).

A nine-camera optical motion tracking system (Vicon, Oxford, United Kingdom) was used to obtain kinematic data. Retro-reflective markers (14 mm diameter) were secured to the skin on the incisura jugularis (IJ), xiphoid process (PX), seventh cervical vertebra (C7), and eighth thoracic vertebra (T8) (Fig. 1). Clusters of three markers were affixed over the spine of the scapula (Prinold et al., 2011) and on the upper arm, just below the insertion of the deltoid, on the dominant side. Coordinate frames for the thorax, scapula, and upper arm were defined as recommended by the International Society of Biomechanics (Wu et al., 2005). The coordinate frame of the scapula was established with the arms at 90° of elevation in the coronal plane (Shaheen et al., 2011). Additional markers were placed on the shoulders and hands, providing participants with visual feedback from the motion capture system to assist them in performing each planar movement.

Participants performed maximal elevation and depression in the coronal, scapular, and sagittal planes with both arms simultaneously, using a metronome to maintain an average velocity of approximately 160°/s. Participants were instructed to perform each motion with wrist in a neutral position and the thumb pointing superiorly. Participants were permitted to practise the movements before recording the kinematics. Between six and eight repetitions were performed and five consecutive cycles from the middle of the trial were selected for analysis.

### 2.3 Data analysis

Raw data were twice filtered with a second-order Butterworth filter (Thigpen et al., 2010, Winter et al., 1974) and, following a frequency analysis (Angeloni et al., 1994), filtered with a cut-off of 5 Hz. The glenohumeral joint CoR was calculated with the Gamage and Lasenby (2002) algorithm using the clusters of markers on the scapula and upper arm. The shoulder complex CoR was determined by finding the instantaneous helical axis (IHA) (Reichl and Auzinger, 2012, Woltring et al., 1985) in the thorax technical coordinate system (TCS) using custom-written code (MATLAB, MathWorks, Natick, USA). Because the method is sensitive to low angular velocities, the IHA was calculated when the velocity was greater than 14.3°/s (Stokdijk et al., 2000). The global CoR was found by taking the intersection between the plane of motion and the IHA. Therefore, for each plane of motion, two coordinates were defined the location of the CoR: one horizontal and one vertical. The position of the CoR was interpolated for every 0.5° of elevation between 45° and 100° of humerothoracic elevation. The RoM was limited to take into account the aforementioned velocity requirement for the calculation of the IHA, and because scapular kinematics have been found to be less accurate for angles of elevation over 100° (van Andel et al., 2009).

To compare the CoR between participants, its mean position in the thorax TCS, determined from the five trials, was normalised for each motion. Normalisation was performed using the distances between anatomic landmarks and anthropometric measures scaled from participant height. Four different methods were trialled along the superior–inferior component and the medial–lateral component, resulting in normalising distances of *D_y_* and *D_z_*, respectively (Table 1 and Fig. 1). For the anterior–posterior axis, two distances (*D_x_*) were used. The location of the CoR was defined relative to C7 and was determined by three coefficients, *A*, *B*, and *C*, where CoR*_x_*=*A*∙*D_x_*; CoR*_y_*=*B*∙*D_y_*; CoR*_z_*=*C*∙*D_z_*. The distance (Delta) between the mean CoR and the instantaneous CoR was then calculated for each 0.5° of elevation. Two-way repeated-measures Analyses of Variance (ANOVAs) were used to compare the Deltas of each coordinate direction for angles of elevation of 45°, 60°, 70°, 80°, 90° and 100° in elevation and depression, with the first factor being the method of normalisation and the second the angle of elevation. Pairwise comparisons were performed with a Bonferroni correction for multiple comparisons. Statistical significance was set at an alpha level 0.05.

*S*_intra_ for each plane of motion was determined from the mean of the repeatability coefficient (Vaz et al., 2013) of the CoR coordinates in the thorax TCS across the five repetitions, for all participants, over all angles. *S*_inter_ for each plane of motion was found from the repeatability coefficient of the mean position of the normalised CoR, across all participants. To quantify the effect of the angle of elevation, the smallest ellipse containing the CoRs for all participants, normalised using the preferred approach, was determined for elevation and depression in each plane of motion for the full RoM, the lower portion of the RoM (45−70°), and the upper portion of the RoM (70−100°). The split between lower and upper portions was selected to align with the change in scapulohumeral rhythm at 70° of unloaded humeral elevation (Kon et al., 2008).

## 3 Results

### 3.1 Influence of the method of normalisation

**Fig. 2:**
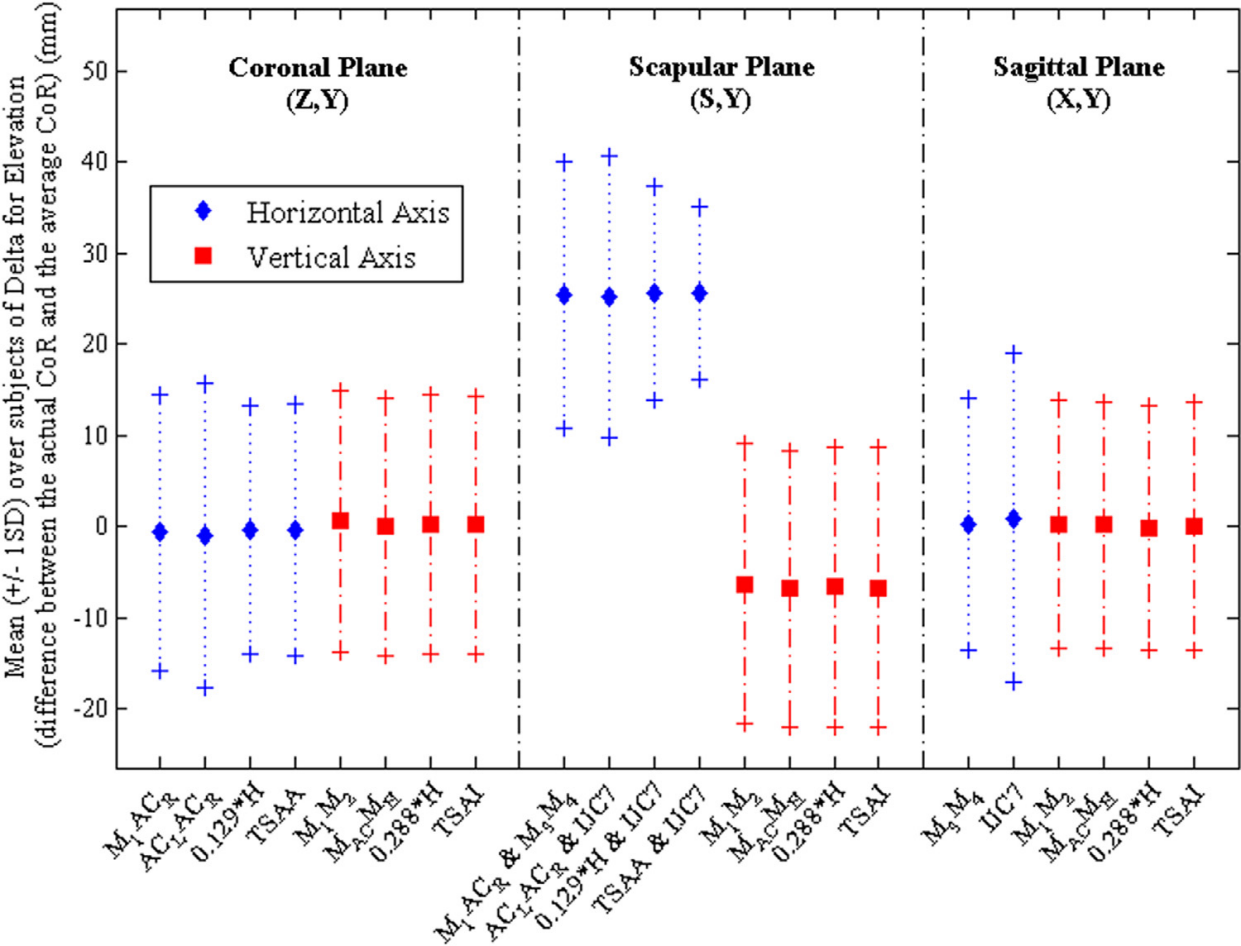
Mean (±1SD) of the distance (Delta) between the mean CoR and the instantaneous CoR, for the three planes of elevation, for the elevation phase, using the methods of normalisation described in Table 1.

**Fig. 3:**
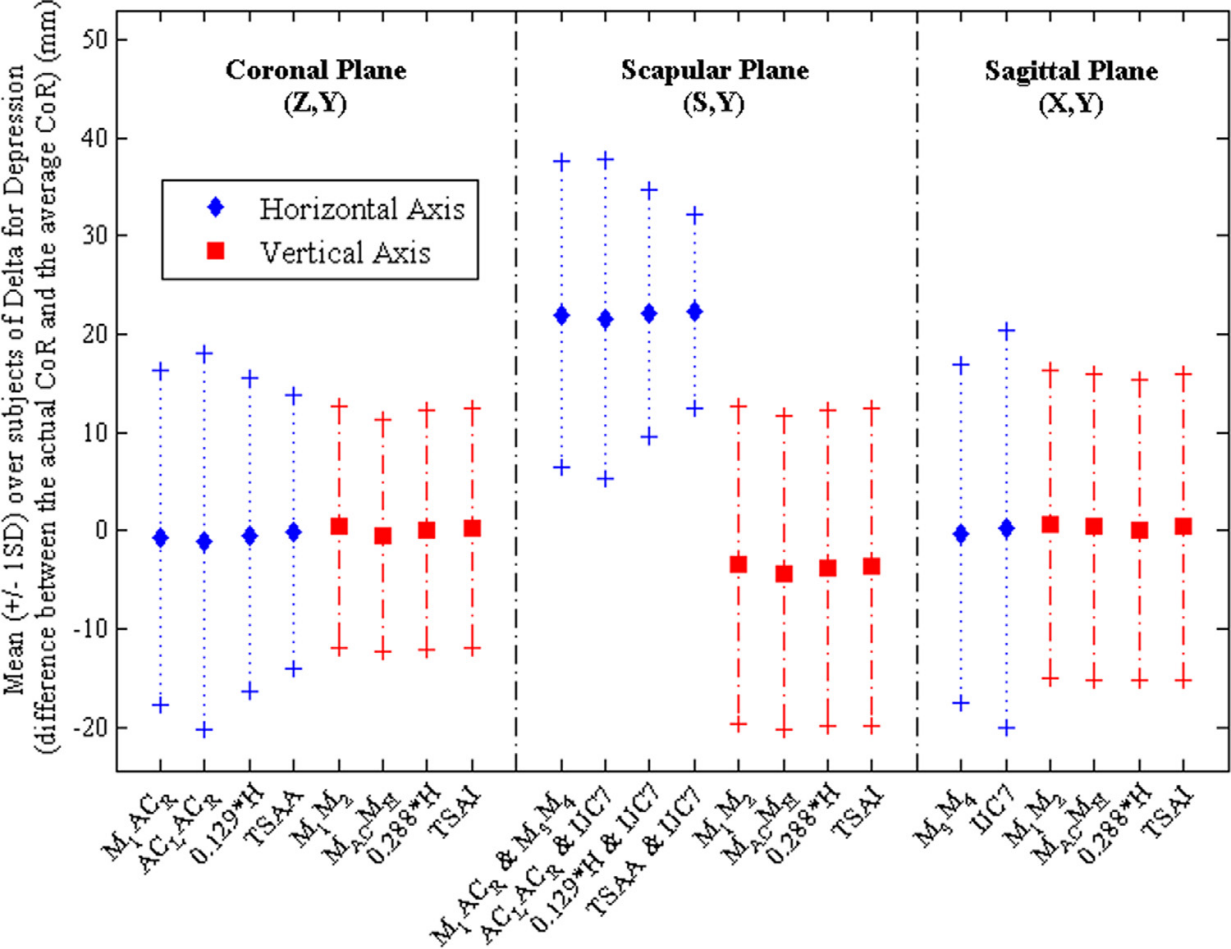
Mean (±1SD) of the distance (Delta) between the mean CoR and the instantaneous CoR, for the three planes of elevation, for the depression phase, using the methods of normalisation described in Table 1.

There was no statistically significant two-way interaction between angle and normalisation (*F*_3,165_=0, *p*=1). No significant differences were found between methods of normalisation when Deltas were compared (0.01<*F*_3,165_<0.06, *p*>0.98; Fig. 2, Fig. 3). However, differences were found across the angles of elevation (2.71<*F*_3,165_<20.38, *p*<0.03) for all planes of motion, except the horizontal component in the scapular plane for the depression phase (*F*_3,165_=1.13, *p*=0.346), and the vertical component in the scapular plane for the elevation phase (*F*_3,165_=2.18; *p*=0.057).

As no differences were observed between normalisation methods, the preferred normalisation dimension was selected as that which provided the smallest Delta for the largest number of individual participants. These dimensions were: the distance between the midpoint of IJ and PX (M_3_) and the midpoint between C7 and T8 (M_4_) for *X*, the distance between the midpoint of the left and right acromioclavicular joints (M_AC_) and the midpoint between the two anterior superior iliac spines (M_H_) for *Y*, and 0.129 times the height of the participant for *Z* (Winter, 2009). The values of the coefficients *A*, *B*, and *C* using the preferred normalisation dimensions are presented in Table 2 for elevation and depression in each plane of elevation.

### 3.2 Repeatability

Across all motions, *S*_intra_ and *S*_inter_ did not exceed 13 mm and 11%, respectively, for the horizontal coordinate and 10 mm and 4%, respectively, for the vertical coordinate (Table 3).

### 3.3 Variation with angle of elevation

**Fig. 4:**
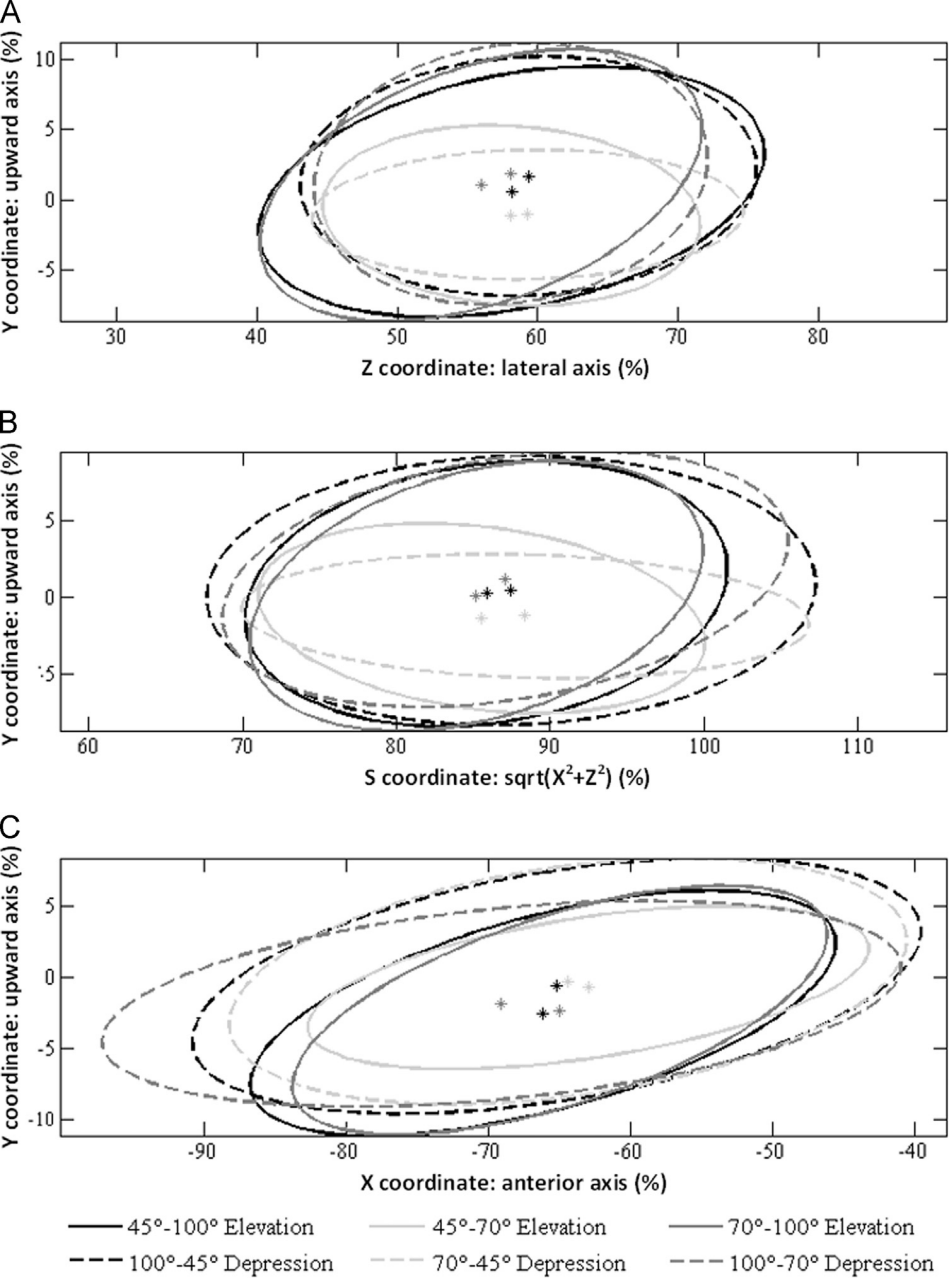
Ellipses containing the normalised locations of the centres of rotation for all subjects for motions in the (A) coronal, (B) scapular, and (C) sagittal planes for the full RoM, the lower portion of the RoM (45−70°), and the upper portion of the RoM (70−100°).

The locations of the ellipses containing the CoRs for each plane of motion in elevation and depression were similar across participants. Maximum variation of the CoR׳s position with the angle of elevation was 20 mm for all planes. The dimensions of the ellipses were largely similar, with the exception of those for the lower portion of the RoM in the coronal and scapular planes, which had a smaller vertical axis, indicating less movement of the CoR (Fig. 4).

## 4 Discussion

While normalised regression equations have been used to determine the location of the CoRs of other joints, such as the hip, in relation to anatomic landmarks (Harrington et al., 2007), this is the first such attempt for the shoulder complex. It has been found previously that the error in locating landmarks by palpation is on the order of 1 cm (Barnett et al., 1999, Johnson et al., 1993); therefore, the intra-subject repeatability of the CoR determined in this study of less than 13 mm is on the same order as that of locating other anatomic features.

By measuring from the palpated location of C7, we propose a subject-specific method of locating the CoR. This may be employed to allow more effective use of isokinetic dynamometers and other tools for shoulder rehabilitation by aligning the axis of motion with the calculated CoR. To minimise motion of the CoR, limiting motion to the lower elevation angles in the coronal and scapular planes is recommended. As the centres and dimensions of the ellipses that include all positions of the CoR over the whole RoM showed no differences between elevation and depression when considering all participants and all planes, it appears that the same point may be employed for elevation and depression in each plane of motion, although they were considered separately for the purposes of data analysis.

One caveat of this work is that although the CoR coordinates for three common planes of motion of the shoulder have been provided, these cannot be generalised to other, more complex motions. Limitations include the variation in the participants’ ability to perform the motions at the requested velocity; when strongly focusing on maintaining the correct velocity some participants may not have completed their maximal RoM. Future work in this area could investigate the influence of confounding factors, such as the velocity of the movement, the addition of external load and the influence of shoulder conditions or specialist functions, such as over-headed sport, including a comparison between shoulders for those who practice unilateral activities.

## 5 Conclusion

This study has succeeded in identifying a CoR for the shoulder complex for movement in each of the coronal, scapular and sagittal planes. Methods of normalisation were compared. When conducting motion in the coronal, scapular, and sagittal planes, respectively, the coefficients for locating the CoRs of the shoulder complex are −61% (from −79% to −50%), −61% (from −79% to −50%) and −65% (from −79% to −49%) of the vector between the midpoint of IJ and PX (M3) and the midpoint between C7 and T8 (M4); 0% (from −6% to 4%), −1% (from −6% to 4%), and −2% (from −7% to 4%) of the vector between the midpoint of the left and right acromioclavicular joints (MAC) and the midpoint between the two anterior superior iliac spines (MH); and 57% (from 47% to 69%), 57% (from 47% to 69%), and 78% (from 69% to 89%) of 0.129 times the height of the participant in *X*, *Y*, and *Z*. This location should be used for exercises that require the hand to move with a fixed radius. Our findings will allow improvements in the design of exercises and equipment for rehabilitation of the shoulder.

## Conflict of interest statement

The authors have no conflicts of interest to declare.

## Figures and Tables

**Table 1:** Distances used for the four methods of normalisation along the three anatomic axes (*D_x_*, *D_y_*, and *D_z_*). These were calculated using the locations of the incisura jugularis (IJ), xiphoid process (PX), seventh cervical vertebra (C7), eighth thoracic vertebra (T8), midpoint between the IJ and C7 (M_1_), midpoint between the PX and T8 (M_2_), midpoint between IJ and PX (M_3_), midpoint between C7 and T8 (M_4_), left acromioclavicular joint (AC_L_), right acromioclavicular joint (AC_R_), midpoint between AC_L_ and AC_R_ (M_AC_), midpoint between the two anterior superior iliac spines (M_H_), height of the subject (H), the trigonum spinae scapulae (TS), the angulus inferior of the scapulae (AI), the angulus acromialis (AA).

*D_x_*	M_3_M_4_	IJC7		
*D_y_*	M_1_M_2_	M_AC_M_H_	0.288*H	TSAI
*D_z_*	M_1_AC_R_	AC_L_AC_R_	0.129*H	TSAA


**Table 2:** Coefficients *A*, *B*, and *C* for elevation and depression for each plane of motion.

	**Coronal plane**			**Scapular plane**			**Sagittal plane**		
	***A***	***B***	***C***	***A***	***B***	***C***	***A***	***B***	***C***
Elevation	−0.61	0	0.56	−0.61	0	0.56	−0.63	−0.02	0.78
Depression	−0.61	−0.01	0.57	−0.61	−0.01	0.57	−0.66	−0.02	0.78

**Table 3:** Mean (±1SD) of the intra-subject repeatability (*S*_intra_ in mm) and of the inter-subject repeatability (*S*_inter_ in %) for the three planes of motion.

	Phase	Coronal plane		Scapular plane		Sagittal plane	
		Horizontal (mm)	Vertical (mm)	Horizontal (mm)	Vertical (mm)	Horizontal (mm)	Vertical (mm)
*S*_intra_	Elevation	10.3 (5.2)	6.8 (3.6)	2.7 (0.9)	7.3 (2.0)	12.5 (5.6)	11.8 (5.4)
Depression	12.2 (4.3)	7.6 (2.6)	3.1 (1.3)	9.2 (3.4)	16.0 (6.2)	11.7 (3.7)

		Horizontal (%)	Vertical (%)	Horizontal (%)	Vertical (%)	Horizontal (%)	Vertical (%)

*S*_inter_	Elevation	5.2	2.5	5.6	2.6	6.5	2.4
Depression	6.3	2.0	5.8	2.9	8.9	3.1
